# Black Patients with Metastatic Castrate-Resistant Prostate Cancer Have a Shorter Time Interval Between PSA and Clinical Progression on Novel Hormonal Therapies plus Avelumab

**DOI:** 10.1093/oncolo/oyac203

**Published:** 2022-10-09

**Authors:** Charlotte (Manogue) Hawkins, Pedro C Barata, Patrick Cotogno, Gaynelle Davis, Ellen Jaeger, Elisa Ledet, Patrick Miller, Brian Lewis, Oliver Sartor, Jodi Layton

**Affiliations:** Tulane Office of Clinical Research, Tulane School of Medicine, New Orleans, LA, USA; Tulane School of Medicine, New Orleans, LA, USA; University Hospitals Seidman Cancer Center, Cleveland, OH, USA; Tulane Office of Clinical Research, Tulane School of Medicine, New Orleans, LA, USA; Tulane Office of Clinical Research, Tulane School of Medicine, New Orleans, LA, USA; Tulane Office of Clinical Research, Tulane School of Medicine, New Orleans, LA, USA; Tulane Office of Clinical Research, Tulane School of Medicine, New Orleans, LA, USA; Tulane Office of Clinical Research, Tulane School of Medicine, New Orleans, LA, USA; Tulane School of Medicine, New Orleans, LA, USA; Tulane School of Medicine, New Orleans, LA, USA; Tulane School of Medicine, New Orleans, LA, USA

**Keywords:** metastatic prostate cancer, avelumab, Black/African American, metastatic castrate-resistant prostate cancer

## Abstract

**Background:**

Black men are at higher risk for prostate cancer death. Previous studies showed a benefit of different therapies, including immune-based therapy, for Black men with metastatic prostate cancer. We sought to explore the efficacy of the PD-L1 inhibitor avelumab in Black men with metastatic castrate-resistant prostate cancer (mCRPC) progressing after abiraterone or enzalutamide.

**Methods:**

This pilot phase II study enrolled self-identified Black patients who developed mCRPC on next-generation hormonal therapies (NHTs) abiraterone acetate or enzalutamide (NCT03770455). Enrolled patients received avelumab 10mg/kg IV every 2 weeks while remaining on the same NHTs. The primary endpoint of our study was ≥ 50% reduction in prostate specific antigen (PSA) at ≥8 weeks.

**Results:**

A total of eight patients were enrolled. The median duration on NHTs prior to enrollment was 364 days (95% CI, 260.9-467.1). The median time to initiate avelumab was 8 days (3-14). With a median follow-up of 196 days, no patients achieved the primary endpoint. The median time to PSA progression was 35 days (95 CI%, 0-94.8) and the median time to radiographic and/or clinical progression was 44 days (95 CI%, 0-118.5). The study was closed prematurely due to safety concerns related to the rapid clinical progression observed in the patients enrolled on study.

**Conclusion:**

In conclusion, the addition of avelumab to NHT did not demonstrate clinical activity in Black men with new mCRPC. The unexpected short interval between PSA and radiographic and/or clinical progression observed in this study has potential clinical implications.

ClinicalTrials.gov Identifier: NCT03770455 (IND number 139559).

## Teaser

Previous studies have shown the benefit of immune-based therapies, such as sipuleucel-T, for Black men with metastatic prostate cancer. This study explored the efficacy of the PD-L1 inhibitor avelumab in Black men with metastatic castrate-resistant prostate cancer progressing after abiraterone or enzalutamide.

**Lessons Learned**

- The addition of avelumab did not demonstrate clinical activity in a cohort of Black men with metastatic castrate-resistant prostate cancer (mCRPC) that had progressed on abiraterone acetate or enzalutamide.
- Black men with mCRPC had a short interval between PSA and radiographic/clinical progression on this trial.

## Discussion

**Figure 1.**
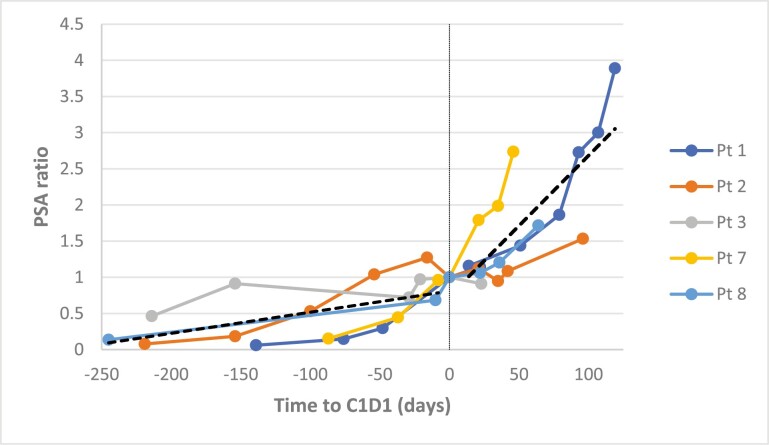
PSA kinetics of enrolled patients. The accelerated PSA progression after C1D1 compared with prior to C1D1 is represented by linear trend lines. PSA ratio was calculated by PSA at timepoint divided by PSA at day 0 (range, days –10 to 0).

Our group and others have previously shown that immune checkpoint inhibitors are effective in specific prostatic tumors with microsatellite instability and high tumor mutational burden but their effect in the biology of prostate cancer is not entirely known.^1,2^ In the present study, avelumab had no activity in an unselected group of Black patients with mCRPC. Furthermore, the interval seen in this trial between trial entry and PSA progression [35 days (95 CI%, 0-94.8)], compared with radiographic and clinical progression [44 days (95 CI%, 0-118.5)] was quite short (Fig. 1 provides more details on PSA progression). There was no activity of avelumab detected in this setting.

In both COU-AA-302 (abiraterone acetate) and PREVAIL (enzalutamide) which enrolled mainly Caucasian patients, the time interval between PSA progression and radiographic progression was approximately 5.3 and 5.1 months, respectively.^3,4^ In a trial that enrolled both Black men and Caucasians, George et al reported that Caucasian and Black men had a discrepancy in their time between PSA progression and radiographic progression. While Caucasians had a 5.7 month time interval between PSA and radiographic progression, Black men had a nearly identical PSA and radiographic progression.^5^

As we expand clinical trial access and accrual to Black men, these results suggest that the interval between PSA and radiographic progression in Black men is likely to be short and thus more frequent radiographic monitoring might be necessary. Of note, this study was designed in the pre-PET scan era. Ongoing studies with PET imaging using fluciclovine or PSMA tracers (eg, NCT04158245) may offer an opportunity to improve response monitoring allowing for more effective and timely treatment decisions.

**Table:** 

Trial Information	
Disease	Prostate cancer
Stage of disease/treatment	Metastatic castrate resistant
Prior therapy	Prior therapy with novel hormonal agents
Type of study	Phase II, investigator-initiated study
Primary endpoint	The primary endpoint was PSA response defined as ≥ 50% reduction in PSA at ≥8 weeks
Secondary endpoints	PSA progression-free survival using PCWG3 criteria; radiographic PFS (rPFS) by PCWG3 criteria, overall survival (OS), and safety as determined by CTCAE v4.3 criteria
Investigator’s analysis	Terminated due to safety concerns with rapid progression in this setting

**Table:** 

Drug Information	
Generic/working name	Avelumab
Company name	EMD Serono: a Merck company
Drug type	Immunotherapy; checkpoint inhibitor
Drug class	Monoclonal antibody
Dose	10
Unit	mg/kg
Route	Intravenous (i.v.)
Schedule of administration	Every 2 weeks

**Table:** 

Patient Characteristics	
Number of patients, male	6
Number of patients, female	Not applicable
Stage	IV
Age: median(range)	62 (54-73) years
Number of prior systemic therapies: median(range)	1 (per study design)
Performance status: ECOG	0: 40% (2/5) 1: 60% (3/5)
Gleason 8-10 at diagnosis	60%

**Table:** 

Primary Assessment Method	
Number of patients screened	8
Number of patients enrolled	6
Number of patients evaluable for toxicity	6
Number of patients evaluated for efficacy	5
Evaluation method	RECIST 1.1, Tumor Marker (PSA), PCWG3 criteria
Outcome notes	The median time between study enrollment and C1D1 of avelumab was 8 days (3-14). With a median follow-up of 196 days, the median number of avelumab doses was 3 (1-9). One patient withdrew consent after one avelumab dose and was not included in the efficacy analysis. PSA responses ≥ 50% (primary endpoint) were not observed in any patient, although one patient had a PSA decline (25%). The median time to PSA progression was 35 days (95 CI%, 0-94.8). Figure 1 represents the PSA kinetics of patients enrolled in this study showing an accelerated phase after C1D1 compared with that prior to avelumab initiation. The median time to radiographic and clinical progression was 44 days (95 CI%, 0-118.5). Furthermore, all patients had evidence ofclinical progression that included cord compression (*n* = 1), pathologic bone fracture (*n* = 1), pelvic mass with rectal invasion (*n* = 1) and cancer-related pain requiring palliative radiation therapy (*n* = 1). In 2 cases, clinical progression occurred within 8 weeks of starting avelumab. There were no new avelumab-related adverse events. Two patients were able to receive subsequent systemic therapy with taxane-based chemotherapy while one patient underwent best supportive care; one patient was lost to follow up. The primary endpoint was not met, and due to the lack of efficacy and the high rate of rapid clinical progression, the study was closed prematurely.

## Assessment, Analysis, and Discussion

**Table:** 

Completion	Study terminated prior to completion
Investigator’s assessment	Level of activity did not meet planned endpoint

Black men are known to be at higher risk for prostate cancer death.^6^ Paradoxically, several studies have shown that Black patients with metastatic prostate cancer have favorable outcomes with novel hormonal therapies (NHTs), taxane-based chemotherapy and immunotherapy Sipuleucel-T compared with non-Hispanic White men.^5,7,8^ Immune checkpoint inhibitors are effective for patients with various cancers but have shown limited efficacy in unselected patients with mCRPC.

Black patients are continually underrepresented in clinical trials and there are possible racial differences in the molecular profiling of tumors and outcomes of these patients that require further investigation of active therapies in this underrepresented group.^10^ Thus, we launched a pilot, single site investigator-initiated phase II study to explore the efficacy of avelumab, an immune checkpoint inhibitor against PD-L1, in self-identified Black men with mCRPC with serologic progression on NHT (IND number 139559; NCT03770455). From January 2019 to December 2020, a total of 8 patients underwent screening and 6 patients were eligible and enrolled in this study.

At the time of trial design, immune checkpoint inhibitors were being actively testing in several solid tumors and specific data on prostatic malignancies such as the phase III trial KEYNOTE-199 (NCT02787005) was not available. Results from KEYNOTE 199 published in 2019, showed the limited activity of immunotherapy unselected group of mCRPC patients, however distribution by race has not been reported.^9^ Pembrolizumab approval remains the only immune checkpoint inhibitor available for prostate cancer, with a specific requirement for tumors with mismatch repair defects, microsatellite instability or high tumor mutational burden.

By the contrary, hyperprogression—defined as a paradoxical boost in tumor growth—has been observed in 4%-29% of solid tumors under treatment with immune checkpoint inhibitors.^11^ In our study, all enrolled patients had PSA progression at time of study entry per inclusion criteria, however we observed an accelerated PSA progression after starting avelumab (C1D1) compared with PSA kinetics prior to C1D1, as shown in Fig. 1. These findings in addition to clinical deterioration of the patients, led to the premature closure of the study.

Our study did not require specific genomic alterations for eligibility, but molecular profiling was required at baseline for all patients. Notably, none of the enrolled patients had known immune-related markers that predict response to immune checkpoint inhibitors. One patient with a pathogenic somatic mutation in the homologous recombination repair gene FANCA had no response to avelumab, yet there is prostate cancer data suggesting other homologous recombination repair gene defects might be predictive of response to immune checkpoint inhibitors, but further validation is required.^12^

Critically, we were concerned at the number of serious adverse events experienced by this population, not related to treatment but with progression of their disease. Patients experienced clinical progression with serious events at a much more rapid rate than expected for men of their disease burden and stage of disease. Collectively, our group decided to close the study for safety concerns due to rapid progression of enrolled patients. Further investigation is necessary to confirm these racial differences and better inform the handling of treatment and expectations for Black men with mCRPC.

## Data Availability

The data underlying this article will be shared on reasonable request to the corresponding author.
